# Complete Genome Sequence of a Novel Reassortant H6N8 Avian Influenza Virus Isolated from Wild Waterfowl in Poyang Lake, China

**DOI:** 10.1128/genomeA.01542-16

**Published:** 2017-02-02

**Authors:** Guangyu Hou, Jinping Li, Cheng Peng, Suchun Wang, Jiming Chen, Wenming Jiang

**Affiliations:** China Animal Health and Epidemiology Center, Qingdao, China

## Abstract

Here, we report the complete genome sequence of an H6N8 avian influenza virus (AIV) isolated from wild waterfowl in Poyang Lake, China, in 2016. Phylogenetic analysis showed that it was a novel reassortant AIV between domestic ducks and wild waterfowl. The finding of this study is helpful for our understanding of the ecology and the evolutionary characteristics of H6 subtypes of AIV in birds.

## GENOME ANNOUNCEMENT

Wild birds, especially waterfowl, are thought to be the natural reservoirs of avian influenza viruses (AIVs) (1) and are known to play a major role in the evolution, maintenance, and spread of AIVs. Migratory birds infected with an AIV may spread the virus into domestic poultry and undergo wide reassortment with the AIVs circulating in poultry. Influenza viruses of the H6 subtype have been isolated from wild and domestic aquatic and terrestrial avian species throughout the world since their first detection in 1965. Since 1997, H6 viruses have been detected frequently in the live poultry markets of southern China.

In this study, a novel recombinant H6N8 subtype AIV, named A/wild bird/Jiangxi/P419/2016(H6N8), was isolated from the feces of wild waterfowl in Poyang Lake, Jiangxi, China, in January 2016. Here, we amplified and analyzed the complete genome sequence of the strain to investigate its genetic characterization (2). The full genome of the strain was sequenced with an ABI 3730xl DNA analyzer.

The complete genome of the strain consists of eight segments, encoding PB2, PB1, PA, HA, NP, NA, M, and NS proteins, with corresponding nucleotide (nt) lengths of 2,341, 2,341, 2,233, 1,738, 1,565, 1,467, 1,027, and 890 nt, respectively. The amino acid sequence at the cleavage site of the HA gene is PQIETR/GLF, which is typical for low-pathogenicity AIVs (3). The amino acid residues at the receptor binding site in the HA protein are Q226 and G228 (H3 numbering), which indicates its avian-like receptor binding preference (4). The strain has seven potential N-glycosylation sites at positions 26, 27, 39, 306, 311, 498, and 557 in the HA protein, and seven sites at positions 46, 54, 67, 84, 144, 293, and 398 in the NA protein. The PB2 protein possessed E627 and D701, which indicated that the virus was of avian origin (5). Furthermore, there were no deletions in the NA and NS genes and no mutation in the NA and M2 genes, which is associated with resistance to amantadine and rimantadine.

Phylogenetic analysis revealed that the PB2, PB1, PA, HA, NP, NA, and NS genes of the virus were derived from Eurasian lineages but that the M gene was derived from North American lineages. Sequences analysis showed that the HA and NA genes showed the highest sequence homologies (98%) with those of the virus strains A/aquatic bird/Korea/CN20/2009(H6N1) and A/duck/Zhejiang/D1-6/2013(H3N8), respectively. The internal genes showed high nucleotide identity with those of the viruses circulating in wild birds and domestic poultry, including H9N2, H11N2, H12N8, H3N8, H14N3, and H1N2.

In conclusion, the novel H6N8 AIV underwent wide reassortment between the viruses circulating in domestic poultry and wild birds. Our results are useful for understanding the role of migratory birds in exchanging AIV genes between North American and Eurasian lineages and highlight the genetic reassortment between viruses of different subtypes and the evolution of AIVs in China.

### Accession number(s).

The complete genome sequences of A/wild bird/Jiangxi/P419/2016(H6N8) have been deposited in GenBank under accession numbers KX867854 to KX867861.
